# Photo-acoustic dual-frequency comb spectroscopy

**DOI:** 10.1038/s41467-020-17908-9

**Published:** 2020-08-20

**Authors:** Thibault Wildi, Thibault Voumard, Victor Brasch, Gürkan Yilmaz, Tobias Herr

**Affiliations:** 1 Swiss Center for Electronics and Microtechnology (CSEM), Rue de l’Observatoire 58, 2000 Neuchâtel, Switzerland; 2 Center for Free-Electron Laser Science (CFEL), Deutsches Elektronen-Synchrotron (DESY), Notkestr. 85, 22607 Hamburg, Germany

**Keywords:** Optical spectroscopy, Frequency combs, Photoacoustics

## Abstract

Photo-acoustic spectroscopy (PAS) is one of the most sensitive non-destructive analysis techniques for gases, fluids and solids. It can operate background-free at any wavelength and is applicable to microscopic and even non-transparent samples. Extension of PAS to broadband wavelength coverage is a powerful tool, though challenging to implement without sacrifice of wavelength resolution and acquisition speed. Here we show that dual-frequency comb spectroscopy (DCS) and its potential for unmatched precision, speed and wavelength coverage can be combined with the advantages of photo-acoustic detection. Acoustic wave interferograms are generated in the sample by dual-comb absorption and detected by a microphone. As an example, weak gas absorption features are precisely and rapidly sampled; long-term coherent averaging further increases the sensitivity. This novel approach of dual-frequency comb photo-acoustic spectroscopy (DCPAS) generates unprecedented opportunities for rapid and sensitive multi-species molecular analysis across all wavelengths of light.

## Abstract (web summary)

Here, the authors show that the resolution and speed limitations in broadband photo-acoustic spectroscopy can be overcome by combining dual-comb spectroscopy with photo-acoustic detection. This enables broadband detection and allows for rapid and sensitive multi-species molecular analysis across all wavelengths of light.

## Introduction

**Fig. 1:**
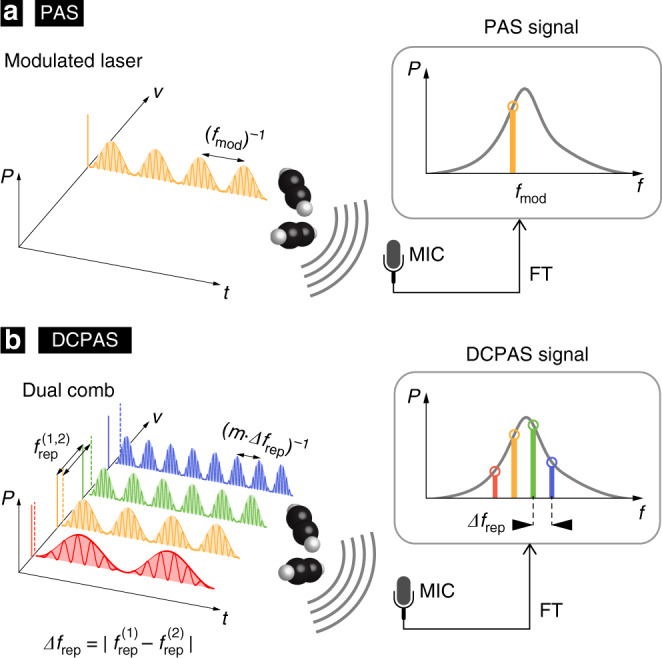
Dual-frequency comb photo-acoustic spectroscopy. **a** In photo-acoustic spectroscopy (PAS), absorption of a modulated laser results in acoustic waves that are recorded by a microphone (MIC). The acoustic spectrum (after Fourier transformation, FT) contains the PAS signal tone at the modulation frequency ${f}_{{\rm{mod}}}$ that indicates the strength of the optical absorption. **b** Dual-frequency comb photo-acoustic spectroscopy (DCPAS) uses broadband dual-frequency combs, whose repetition rates ${f}_{{\rm{rep}}}^{(1)}$ and ${f}_{{\rm{rep}}}^{(2)}$ differ by a small amount *Δf*_rep_. The DCPAS signal is comprised of multiple heterodyne acoustic tones that simultaneously sample the optical absorption spectrum at multiple optical frequencies. (*P*: power; *ν* and *f*: optical and acoustic frequencies; *t*: time).

In PAS^1–4^, optical absorption of a modulated light source leads to periodic heating of a sample and the generation of an acoustic wave that can be detected by a microphone or an equivalent transducer (Fig. 1a). As the detection relies on the acoustic waves (rather than a weak attenuation of an optical signal), photo-acoustic detection can be background-free, with high signal-to-noise ratio (SNR), and importantly, works at any wavelength of light. These unique properties have established PAS in environmental studies, solid state physics, chemical process control, medical application and life science, including for instance absorption measurements in atto-liter droplets^5^, real-time monitoring of an ant’s respiration^6^ and in-vivo tomographic imaging^7^. Quartz-enhanced photo-acoustic spectroscopy (QEPAS)^8–10^ and cantilever-enhanced photo-acoustic spectroscopy (CEPAS)^11,12^ have enabled ultra-sensitive trace gas detection below the part-per-trillion-level^13,14^.

Usually, PAS is performed at one single probing laser wavelength. This is not ideal for the study of multiple species or studies in the presence of uncontrolled background absorption. Multiple laser sources can alleviate this problem to some extent, however, remain constraint to specific use cases. Therefore, in order to achieve broadband wavelength coverage, photo-acoustic detection has been combined with Fourier-transform infrared spectrometers (FTIR-PAS)^15^. The achievable resolution is determined by the scan range of the interferometer, which can reach several meters for high resolution instruments. In addition to temporally incoherent light sources, such as supercontinua^16^, coherent broadband spectra (unresolved optical frequency combs) have been used to improve the overall performance^17,18^. Combining frequency combs with scanning Fourier-transform spectrometers also permits using techniques for sub-nominal resolution^19^. As such, FTIR-PAS represents a powerful tool for broadband photo-acoustic spectroscopy. However, high-resolution FTIR-PAS relies on long mechanical scans, which can limit the acquisition speed and require mechanically stable setups.

Here, we show that the resolution and speed limitations in broadband PAS can be overcome by combining the concept of dual-frequency comb spectroscopy (DCS)^20–24^ with photo-acoustic detection resulting in the new technique of dual-frequency comb photo-acoustic spectroscopy (DCPAS). Photo-acoustic dual-comb multi-heterodyne detection enables the rapid and scan-free acquisition of absorption features with high resolution and precision (traceable to the SI-time standard), thereby enabling background-free, broadband spectroscopy of gases, liquids and solids at any wavelength of light.

## Results

### Concept

Figure 1b illustrates the concept of DCPAS. Similar to conventional DCS, two frequency combs are used in our demonstration whose optical frequency components ${\nu }_{n}^{(i)}$ are described by

1 $${\nu }_{n}^{(i)}=n\cdot {f}_{{\rm{rep}}}^{(i)}+{\nu }_{{\rm{0}}}^{(i)},$$

${f}_{{\rm{rep}}}^{(i)}$ and ${\nu }_{{\rm{0}}}^{(i)}$ denote the repetition rate (i.e. the comb line spacing) and the combs’ optical offset frequencies, respectively. The index *i* = 1, 2 distinguishes the two combs, and *n* = 0, ±1, ±2, . .  are the comb line indices. The combs’ repetition rates and offsets differ only by small amounts $\Delta {f}_{{\rm{rep}}}=\left|{f}_{{\rm{rep}}}^{(1)}-{f}_{{\rm{rep}}}^{(2)}\right|\ll {f}_{{\rm{rep}}}^{(1,2)}$, and $\Delta {\nu }_{0}=\left|{\nu }_{{\rm{0}}}^{(1)}-{\nu }_{{\rm{0}}}^{(2)}\right|\ll {f}_{{\rm{rep}}}^{(1,2)}$, so that pairs of optical comb lines ${\nu }_{n}^{(1)}$ and ${\nu }_{n}^{(2)}$ are only separated by acoustic frequencies. When both combs are optically combined, this can be interpreted as a single frequency comb

2 $${\tilde{\nu }}_{n}=n\cdot \frac{1}{2}\left({f}_{{\rm{rep}}}^{(1)}+{f}_{{\rm{rep}}}^{(2)}\right)+\frac{1}{2}\left({\nu }_{{\rm{0}}}^{(1)}+{\nu }_{{\rm{0}}}^{(2)}\right) ,$$

whose *n*^th^ optical line is modulated in optical power according to $1+\cos (2\pi {f}_{n}t+{\phi }_{n})$ with frequency

3 $${f}_{n}=\left|{\nu }_{n}^{(1)}-{\nu }_{n}^{(2)}\right|=n\cdot \Delta {f}_{{\rm{rep}}}+\Delta {\nu }_{0}$$

and a phase *ϕ*_*n*_. Exposing the sample to the dual-combs, it experiences periodic heating with frequency *f*_*n*_ if light at the optical frequency ${\tilde{\nu }}_{n}$ is absorbed. The periodic heating will lead to the generation of heterodyne acoustic waves in function of the absorbed power. Note that different from conventional DCS, the heterodyning does not happen on an external photo-detector, but indeed in and by the sample itself. The superposition of all acoustic waves results in a series of interferograms, each with a duration of $\Delta {f}_{{\rm{rep}}}^{-1}$, that is detectable by a microphone or an equivalent transducer, provided all acoustic frequencies *f*_*n*_ respect the bandwidth limitation of the transducer.

### Setup

Key to our demonstration are dual-frequency combs with high mutual coherence that enable dense packing of the acoustic multi-heterodyne beatnotes *f*_*n*_ within the microphone’s bandwidth. Dual-combs with high mutual coherence have been implemented in various ways based on mode-locked lasers or electro-optic modulation^25–36^, and have also been extended to the infrared molecular fingerprint regime^37–40^.

**Fig. 2:**
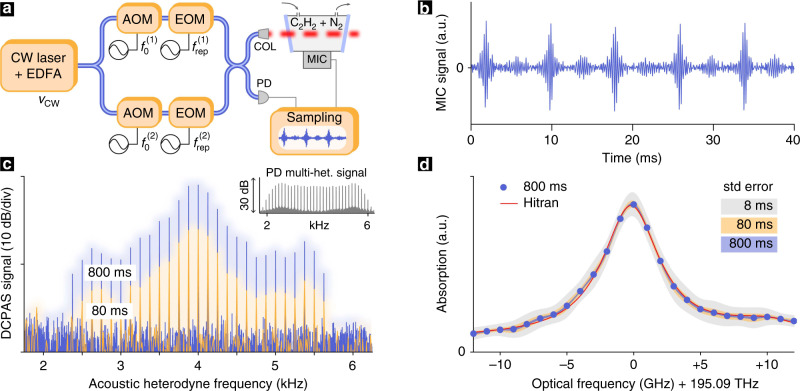
Experimental setup and results. **a** Experimental setup for the photo-acoustic detection of gaseous acetylene (C_2_H_2_). A tunable continuous-wave (CW) laser with optical frequency *ν*_CW_ is amplified by an erbium-doped fiber amplifier (EDFA) and used as a common seed for the generation of two optical frequency combs with repetition rates of ${f}_{{\rm{rep}}}^{(1)}$ and ${f}_{{\rm{rep}}}^{(2)}$ via electro-optic modulation (EOM). Acousto-optic modulation (AOM) of the CW laser with ${f}_{0}^{(1)}$ and ${f}_{0}^{(2)}$ controls the relative offset between both combs. (COL: free space collimator; PD: reference photo-detector MIC: low-noise MEMS microphone, see Methods for more details). **b** Acoustic multi-heterodyne signal recorded by the microphone (5 interferograms; after high-pass filtering) for an acetylene filled cell. **c** Spectrum of the acoustic multi-heterodyne signal for 80 ms and 800 ms long acquisitions. Inset: Multi-heterodyne reference spectrum as recorded by the reference photo-detector (over the same span). **d** Acetylene absorption signature obtained after normalizing the acoustic multi-heterodyne spectrum by the reference spectrum for an 800 ms acquisition duration (blue dots); shaded areas (gray, yellow, blue) represent the standard-error intervals for different acquisition durations (8 ms, 80 ms and 800 ms). The red line shows the HITRAN model for comparison.

In this proof-of-concept demonstration, we use two near-infrared electro-optic combs as shown in (Fig. 2a) with a tunable central wavelength around 1535 nm and each with approximately 40 comb lines, spaced by ${f}_{{\rm{rep}}}^{(1)}=1$ GHz and ${f}_{{\rm{rep}}}^{(2)}={f}_{{\rm{rep}}}^{(1)}+125$ Hz, respectively. The combsʼ relative central offset is adjusted to *Δν*_0_ = 4 kHz as further explained in the Methods section. Combined, both combs deliver 20 mW of average power for photo-acoustic detection.

The combs are sent through a sample cell (see Methods for details) and an off-the-shelf digital micro-electro-mechanical system (MEMS) microphone with 20 kHz bandwidth is used to record the acoustic signal. The repetition rate difference of Δ*f*_rep_ = 125 Hz was chosen so that all acoustic multi-heterodyne beatnotes would be within 2 to 6 kHz and well within the microphone’s bandwidth. Given the combs’ high mutual coherence (sub-Hz multi-heterodyne beatnotes), we note that more beatnotes (i.e., more optical sampling points) could readily be accommodated by lowering Δ*f*_rep_.

A small fraction of the comb light is sent to a photo-detector that provides a reference for normalization of the photo-acoustic signal and also enables an enhancement of the combs’ mutual coherence in post-processing, as we detail below. In a wavelength regime where suitable photo-detectors may not be available, the reference detector could be implemented by photo-acoustic detection of black-body absorption. Both the microphone as well as the photo-detector signals are sampled and recorded.

### Measurements

As a spectroscopic target we choose acetylene gas (C_2_H_2_) at atmospheric pressure and lab temperature as it provides well-known, precisely defined and interference-free absorption features uniquely suitable for validating the new DCPAS method. In a first experiment, the absorption cell is filled with acetylene gas and probed at 1536.71 nm (spectral line strength of 4.882 × 10^−21^ cm/molecule), giving rise to the heterodyne acoustic interferogram signal shown in Fig. 2b. Figure 2c shows two examples of the heterodyne acoustic spectra after Fourier-transformation^41^ (DCPAS signal) for acquisitions with durations of 80 ms (10 interferograms) and 800 ms (100 interferograms), respectively. As expected, a longer acquisition time yields a higher SNR in the DCPAS signal. The absence of a DCPAS signal below 2.4 and above 5.6 KHz is due to the combined drop in the absorption feature and the comb lines intensity. Indeed, and in contrast to conventional DCS, photo-acoustic multi-heterodyne beatnotes are only generated in spectral regions where light is absorbed. Therefore, the number of photo-acoustic multi-heterodyne beatnotes is generally smaller than the number of comb lines. Although this does not allow for measurement of absolute absorption values without prior calibration, it avoids large (shot noise) background signals that can mask spectrally sparse or weak absorption features in conventional DCS^42^.

In order to retrieve the true absorption profile, the acoustic multi-heterodyne beatnotes are normalized to account for the uneven spectral power envelope of the combs. Here, we accomplish this by dividing the DCPAS signal (Fig. 2c) by the photo-detected multi-heterodyne reference beatnotes (inset in Fig. 2c). The mapping of the acoustic to the optical frequency axis is described by Eqs. (2) and (3), implying a compression factor of $({f}_{{\rm{rep}}}^{(1)}+{f}_{{\rm{rep}}}^{(2)})/(2\Delta {f}_{{\rm{rep}}})\approx 8\times 1{0}^{6}$ between acoustic and optical frequency axes. The resulting C_2_H_2_-absorption signature is shown and compared to the HITRAN model^43,44^ in Fig. 2d: Blue dots show the absorption retrieved from an 800 ms long acquisition and shaded areas (gray, yellow, blue) represent the standard-error intervals for different durations of acquisition (8 ms, 80 ms and 800 ms). Excellent agreement between the HITRAN-model (red line) and the measured absorption profile is achieved, with the smallest residuals (below 3% relative to peak absorption) observed with an 800 ms long acquisition. The gray shaded area indicates that a fast, 8 ms long acquisition (i.e. a single interferogram) is sufficient to retrieve the coarse features of the absorption profile. The spectral resolution for each sampling point is given by the combs’ absolute optical linewidth (here:  ~100 kHz), so that instrumental lineshape effects are negligible (resolution 5 orders of magnitude below the width of the absorption feature). Moreover, the frequency spacing of the sampling points (1.0000000675 GHz) is precisely defined by the mean repetition rate of the two combs (Eq. (2)). Here, the absolute frequency offset of the frequency combs is obtained by aligning the measured absorption feature with the HITRAN model, which is straightforward as the shape of the absorption line is recorded; however, model-independent self-referencing techniques^20,21^ could be used as well.

**Fig. 3:**
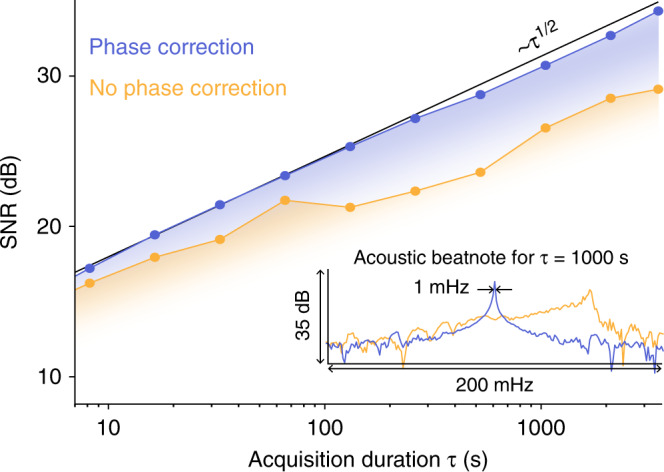
Long-term acquisition. Signal-to-noise ratio (SNR) with (blue) and without (yellow) phase correction as a function of acquisition duration *τ*. The black line indicates the ideal case where the SNR increases proportionally to *τ*^1/2^. Inset: Central heterodyne acoustic beatnote spectrum for *τ* = 1000 s with(blue) and without (yellow) phase correction.

Next, we investigate the extent to which even longer recordings of time *τ* can increase the SNR. To explore this, the cell is filled with N_2_-diluted C_2_H_2_ with a concentration of 1% and probed by combs centered at 1532.83 nm (spectral line strength of 1.035 × 10^−20^ cm/molecule). Acquisitions of different duration are processed (similar to what is shown in Fig. 2) and the SNR of the highest acoustic beatnote (at 4 kHz) is determined as a function of *τ*. Indeed, as Fig. 3 shows, the SNR increases with *τ* (yellow trace), however, it markedly deviates from the *τ*^1/2^-scaling one would expect in a scenario with perfect noise-averaging. This deviation is due to small and slow length fluctuations in the non-common optical path of the combs that limit their mutual coherence on the time scale of few seconds or longer. These slow fluctuations manifest themselves as phase drifts in the multi-heterodyne beatnotes, which fortunately, can easily be tracked and corrected for numerically^26,45–50^. Here, we extract the phase drift (one phase value for all heterodyne beats) from the reference heterodyne signal and, after low-pass filtering (<0.1 Hz), subtract it from the phase of the heterodyne acoustic beatnotes. This a-posteriori phase-correction extends the effective mutual-coherence time of the combs by compensating for the slow path length fluctuations. As shown by the blue trace in Fig. 3, phase correction results in an increase of the SNR close to the ideal scaling (black line) up to the maximal recording duration of 1 hour. This result suggests that even longer recordings could be leveraged to further increase the signal to noise ratio. A small deviation from the ideal scaling is observed for acquisitions longer than 300 s and attributed to residual differential phase drifts between the heterodyne beatnotes, which could be addressed by tracking the phase of each beatnote separately. To further illustrate the effect of phase correction, the inset in Fig. 3 shows a zoom on the central heterodyne acoustic beatnote for a recording time of 1000 s. With phase correction applied (blue trace), a narrow 1 mHz linewidth heterodyne beatnote is detected. Without phase correction (yellow trace) the drifting beatnote has a reduced SNR. Generally, in photo-acoustic spectroscopy, the SNR depends on the used optical power, the absorption coefficient, the photo-acoustic cell design^51^, the microphone, the surrounding matter, environmental conditions (pressure, temperature) as well as the recording duration. In the current proof-of-concept configuration, based on the SNR in Fig. 3, we estimate a minimal detectable noise equivalent C_2_H_2_ concentration of 10 ppm for a recording time of 1000 s. This shows that coherent averaging can also be applied in DCPAS, providing additional opportunities for increasing the sensitivity.

## Discussion

In conclusion, we have demonstrated dual-frequency comb photo-acoustic spectroscopy (DCPAS) as a novel broadband spectroscopic technique that can achieve high resolution, rapid acquisition (here: as short as 8 ms) and sensitive detection. While this demonstration is performed in the near-infrared wavelength range, the concept can readily be translated to any other wavelength range where suitable comb sources are available^21,52^. Therefore, in the mid-infrared and other wavelength regimes where photo-detection is challenging, DCPAS can complement existing DCS approaches (e.g., those based on optical field sampling or up-conversion^53,54^). Importantly, it can also operate on microscopic and even non-transparent samples. In this proof-of-concept demonstration, we have used a very basic photo-acoustic cell design. More elaborate designs with optimized geometries, leveraging optical and acoustic cavity enhancement, could be used to improve the sensitivity^51,55,56^. In addition, more powerful dual-comb laser sources, such as high-power quantum-cascade laser combs^57,58^ could enhance the photo-acoustic signal. Further, broadband dual-comb spectra from mode-locked lasers with high-mutual coherence^25,27^ as well as sensitive multi-MHz bandwidth optical microphones^59,60^, and potentially opto-mechanical transducers^61–63^ could be used to extend the spectral coverage. Particularly for those application where low spectral resolution is sufficient, e.g. in in-vivo hyperspectral tomographic imaging^7^, low-noise high-repetition rate soliton microresonator combs^64–66^ could enable very fast acquisition over a large spectral range. As such, our demonstration generates new opportunities for rapid, sensitive broadband, chemically specific analysis of gases, liquids and solids across all wavelengths of light. The authors would like to make the reader aware of recent parallel work demonstrating the novel method of DCPAS for polymer films^67^, further highlighting the method’s potential as a versatile analysis tool.

## Methods

### Dual-frequency comb source

In order to ensure high mutual coherence between both electro-optic combs, they are derived from a single, free-running continuous-wave (CW) tunable external cavity diode laser with optical frequency *ν*_CW_. Using a free-running laser does not limit the precision of the absorption measurement as the full absorption line profile is recorded. The CW laser is amplified in an erbium-doped fiber amplifier (EDFA) and split into two beams, each traversing first an acousto-optic modulator (AOM) where the laser frequencies are shifted by ${f}_{0}^{(1)}=80$ MHz and ${f}_{0}^{(2)}=80$ MHz + 4 kHz, respectively (i.e., ${\nu }_{0}^{(1,2)}={\nu }_{{\rm{CW}}}+{f}_{0}^{(1,2)}$), to create a relative comb offset of Δ*ν*_0_ = 4 kHz. Next, each beam passes through an electro-optic modulation (EOM) stage that includes one intensity and two phase modulators to generate a series of approximately 40 comb lines, spaced by ${f}_{{\rm{rep}}}^{(1)}=1$ GHz and ${f}_{{\rm{rep}}}^{(2)}={f}_{{\rm{rep}}}^{(1)}+125$ Hz, respectively. All modulation sources are synchronized to a 10 MHz frequency standard to ensure precise sampling and coherence in the acquisition process.

### Sample cell

An aluminum tube (diameter 4 mm, length 10 mm) whose ends are sealed by angled glass windows serves as the photo-acoustic sample cell. An 8-fold multi-pass configuration of the comb light is achieved via two slightly tilted flat mirrors arranged around the cell. Attached to the sidewall of the tube and connected through a small hole is an off-the-shelf digital MEMS microphone (ICS-43434) with a sensitivity of -26 dBFS at 94 dB sound pressure level (SPL) and an equivalent input noise of 30 dBA SPL. A battery-powered amplifier and digitizer is used to record the acoustic signals for a memory limited duration of up to 1 h.

## Supplementary information

Peer Review File

## Data Availability

The data that support the findings of this study are available from the corresponding author upon reasonable request.

## Code availability

The code producing the figures is available from the corresponding author upon reasonable request.

## References

[1] Kreuzer LB, Patel CKN (1971). Nitric oxide air pollution: detection by optoacoustic spectroscopy. Science.

[2] Rosencwaig A (1973). Photoacoustic spectroscopy of biological materials. Science.

[3] Patel CKN, Tam AC (1979). Optoacoustic spectroscopy of liquids. Appl. Phys. Lett..

[4] Li J, Chen W, Yu B (2011). Recent progress on infrared photoacoustic spectroscopy techniques. Appl. Spectrosc. Rev..

[5] Cremer JW, Thaler KM, Haisch C, Signorell R (2016). Photoacoustics of single laser-trapped nanodroplets for the direct observation of nanofocusing in aerosol photokinetics. Nat. Commun..

[6] Herpen MMJWv (2006). Optical parametric oscillator-based photoacoustic detection of CO 2 at 4.23 um allows real-time monitoring of the respiration of small insects. Appl. Phys. B.

[7] Wang X (2003). Noninvasive laser-induced photoacoustic tomography for structural and functional in vivo imaging of the brain. Nat. Biotechnol..

[8] Kosterev AA, Bakhirkin YA, Curl RF, Tittel FK (2002). Quartz-enhanced photoacoustic spectroscopy. Opt. Lett..

[9] Patimisco P, Scamarcio G, Tittel FK, Spagnolo V (2014). Quartz-enhanced photoacoustic spectroscopy: a review. Sensors.

[10] Wu H (2017). Beat frequency quartz-enhanced photoacoustic spectroscopy for fast and calibration-free continuous trace-gas monitoring. Nat. Commun..

[11] Koskinen V, Fonsen J, Roth K, Kauppinen J (2007). Cantilever enhanced photoacoustic detection of carbon dioxide using a tunable diode laser source. Appl. Phys. B.

[12] Peltola J (2013). High sensitivity trace gas detection by cantilever-enhanced photoacoustic spectroscopy using a mid-infrared continuous-wave optical parametric oscillator. Opt. Express.

[13] Spagnolo V (2012). Part-per-trillion level SF_6_ detection using a quartz enhanced photoacoustic spectroscopy-based sensor with single-mode fiber-coupled quantum cascade laser excitation. Opt. Letters.

[14] Tomberg T, Vainio M, Hieta T, Halonen L (2018). Sub-parts-per-trillion level sensitivity in trace gas detection by cantilever-enhanced photo-acoustic spectroscopy. Sci. Rep..

[15] Busse G, Bullemer B (1978). Use of the opto-acoustic effect for rapid scan Fourier spectroscopy. Infrared Phys..

[16] Mikkonen T (2018). Broadband cantilever-enhanced photoacoustic spectroscopy in the mid-IR using a supercontinuum. Opt. Lett..

[17] Sadiek I, Mikkonen T, Vainio M, Toivonen J, Foltynowicz A (2018). Optical frequency comb photoacoustic spectroscopy. Phys. Chem. Chem. Phys..

[18] Karhu J (2019). Broadband photoacoustic spectroscopy of 14CH4 with a high-power mid-infrared optical frequency comb. Opt. Lett..

[19] Maslowski P (2016). Surpassing the path-limited resolution of Fourier-transform spectrometry with frequency combs. Phys. Rev. A.

[20] Coddington I, Newbury N, Swann W (2016). Dual-comb spectroscopy. Optica.

[21] Picqué N, Hänsch TW (2019). Frequency comb spectroscopy. Nat. Photonics.

[22] Schiller S (2002). Spectrometry with frequency combs. Opt. Lett..

[23] Keilmann F, Gohle C, Holzwarth R (2004). Time-domain mid-infrared frequency-comb spectrometer. Opt. Lett..

[24] Schliesser A, Brehm M, Keilmann F, Weide DWvd (2005). Frequency-comb infrared spectrometer for rapid, remote chemical sensing. Opt. Express.

[25] Coddington I (2008). Coherent multiheterodyne spectroscopy using stabilized optical frequency combs. Phys. Rev. Lett..

[26] Zolot AM (2012). Direct-comb molecular spectroscopy with accurate, resolved comb teeth over 43 THz. Opt. Lett..

[27] Okubo S (2015). Ultra-broadband dual-comb spectroscopy across 1.0-1.9 um. Appl. Phys. Express.

[28] Millot G (2016). Frequency-agile dual-comb spectroscopy. Nat. Photonics.

[29] Ideguchi T, Nakamura T, Kobayashi Y, Goda K (2016). Kerr-lens mode-locked bidirectional dual-comb ring laser for broadband dual-comb spectroscopy. Optica.

[30] Mehravar S, Norwood RA, Peyghambarian N, Kieu K (2016). Real-time dual-comb spectroscopy with a free-running bidirectionally mode-locked fiber laser. Appl. Phys. Lett..

[31] Zhao X (2016). Picometer-resolution dual-comb spectroscopy with a free-running fiber laser. Opt. Express.

[32] Link SM, Maas DJHC, Waldburger D, Keller U (2017). Dual-comb spectroscopy of water vapor with a free-running semiconductor disk laser. Science.

[33] Hébert NB, Lancaster DG, Michaud-Belleau V, Chen GY, Genest J (2018). Highly coherent free-running dual-comb chip platform. Opt. Lett..

[34] Chen Z, Yan M, Hänsch TW, Picqué N (2018). A phase-stable dual-comb interferometer. Nat. Commun..

[35] Martín-Mateos P, Jerez B, Largo-Izquierdo P, Acedo P (2018). Frequency accurate coherent electro-optic dual-comb spectroscopy in real-time. Opt. Express.

[36] Gu C (2020). Passive coherent dual-comb spectroscopy based on optical-optical modulation with free running lasers. PhotoniX.

[37] Ycas G (2018). High-coherence mid-infrared dual-comb spectroscopy spanning 2.6 to 5.2 um. Nat. Photonics.

[38] Muraviev AV, Smolski VO, Loparo ZE, Vodopyanov KL (2018). Massively parallel sensing of trace molecules and their isotopologues with broadband subharmonic mid-infrared frequency combs. Nat. Photonics.

[39] Kayes MI, Abdukerim N, Rekik A, Rochette M (2018). Free-running mode-locked laser based dual-comb spectroscopy. Opt. Lett..

[40] Liao R (2018). Dual-comb spectroscopy with a single free-running thulium-doped fiber laser. Opt. Express.

[41] Virtanen P (2020). SciPy 1.0: fundamental algorithms for scientific computing in Python. Nat. Methods.

[42] Newbury NR, Coddington I, Swann W (2010). Sensitivity of coherent dual-comb spectroscopy. Opt. Express.

[43] Kochanov R (2016). HITRAN Application Programming Interface (HAPI): a comprehensive approach to working with spectroscopic data. J. Quant. Spectrosc. Radiat. Transfer.

[44] Gordon I (2017). The HITRAN2016 molecular spectroscopic database. J. Quant. Spectrosc. Radiat. Transfer.

[45] Roy J, Deschênes J-D, Potvin S, Genest J (2012). Continuous real-time correction and averaging for frequency comb interferometry. Opt. Express.

[46] Ideguchi T, Poisson A, Guelachvili G, Picqué N, Hänsch TW (2014). Adaptive real-time dual-comb spectroscopy. Nat. Commun..

[47] Burghoff D, Yang Y, Hu Q (2016). Computational multiheterodyne spectroscopy. Sci. Adv..

[48] Hébert NB (2017). Self-corrected chip-based dual-comb spectrometer. Opt. Express.

[49] Zhu Y (2018). Light emitting diodes based photoacoustic imaging and potential clinical applications. Sci. Rep..

[50] Sterczewski LA, Sterczewski LA, Sterczewski LA, Westberg J, Wysocki G (2019). Computational coherent averaging for free-running dual-comb spectroscopy. Opt. Express.

[51] Bijnen FGC, Reuss J, Harren FJM (1996). Geometrical optimization of a longitudinal resonant photoacoustic cell for sensitive and fast trace gas detection. Rev. Sci. Instrum..

[52] Schliesser A, Picqué N, Hänsch TW (2012). Mid-infrared frequency combs. Nat. Photonics.

[53] Kowligy AS (2019). Infrared electric field sampled frequency comb spectroscopy. Sci. Adv..

[54] Chen, Z., Hänsch, T. W. & Picqué, N., Upconversion mid-infrared dual-comb spectroscopy. Preprint at http://arxiv.org/abs/2003.06930 (2020).

[55] Bernhardt B (2010). Cavity-enhanced dual-comb spectroscopy. Nat. Photonics.

[56] Harren, F. J. & Cristescu, S. M. Photoacoustic spectroscopy in trace gas monitoring. In *Encyclopedia of Analytical Chemistry* 1–29 (2019). 10.1002/9780470027318.a0718.pub3.

[57] Villares G, Hugi A, Blaser S, Faist J (2014). Dual-comb spectroscopy based on quantum-cascade-laser frequency combs. Nat. Commun..

[58] Gianella M (2020). High-resolution and gapless dual comb spectroscopy with current-tuned quantum cascade lasers. Opt. Express.

[59] Rosenthal A, Razansky D, Ntziachristos V (2011). High-sensitivity compact ultrasonic detector based on a pi-phase-shifted fiber Bragg grating. Opt. Lett..

[60] Fischer B (2016). Optical microphone hears ultrasound. Nat. Photonics.

[61] Gavartin E, Verlot P, Kippenberg TJ (2012). A hybrid on-chip optomechanical transducer for ultrasensitive force measurements. Nat. Nanotechnol..

[62] Stiller B, Dainese P, Verhagen E (2019). Optoacoustics—advances in high-frequency optomechanics and Brillouin scattering. APL Photonics.

[63] Mason D, Chen J, Rossi M, Tsaturyan Y, Schliesser A (2019). Continuous force and displacement measurement below the standard quantum limit. Nat. Phys..

[64] Herr T (2014). Temporal solitons in optical microresonators. Nat. Photonics.

[65] Suh M-G, Yang Q-F, Yang KY, Yi X, Vahala KJ (2016). Microresonator soliton dual-comb spectroscopy. Science.

[66] Kippenberg TJ, Gaeta AL, Lipson M, Gorodetsky ML (2018). Dissipative Kerr solitons in optical microresonators. Science.

[67] Friedlein JT (2020). Dual-comb photoacoustic spectroscopy. Nat. Commun..

