# Supplementary material for: Photo-acoustic dual-frequency comb spectroscopy
Source: Nat Commun. 2020 Aug 20;11:4164. doi: 10.1038/s41467-020-17908-9 (PMC7441402; doi:10.1038/s41467-020-17908-9)
Supplement: Supplementary file 1 — Peer Review File [file 41467_2020_17908_MOESM1_ESM.pdf]

Editorial Note: This manuscript has been previously reviewed at another journal that is not operating a transparent peer review scheme. This document only contains reviewer comments and rebuttal letters for versions considered at Nature Communications.

REVIEWERS' COMMENTS:

Reviewer #2 (Remarks to the Author):

The authors have properly addressed the concerns raised in the Nat. Phot. review process and revised the manuscript accordingly. I view the work as a clear demonstration of a novel technique resulting in an important advance in the field and consider it suitable for publication in Nature Communications.

Reviewer #3 (Remarks to the Author):

My concerns have been addressed and I support publication
